# Developmental Splicing Deregulation in Leukodystrophies Related to EIF2B Mutations

**DOI:** 10.1371/journal.pone.0038264

**Published:** 2012-06-21

**Authors:** Aurélia Huyghe, Laetitia Horzinski, Alain Hénaut, Marina Gaillard, Enrico Bertini, Raphael Schiffmann, Diana Rodriguez, Yann Dantal, Odile Boespflug-Tanguy, Anne Fogli

**Affiliations:** 1 Génétique, Reproduction et Développement (GReD) Faculté de Médecine, Clermont-Ferrand, France; 2 Université de Clermont, UFR Médecine, Clermont-Ferrand, France; 3 Systématique, Adaptation, Evolution, CNRS - Université Pierre et Marie Curie, Paris, France; 4 Division of Neuromuscular and Neurodegenerative Disorders, Laboratory of Molecular Medicine, Department of Neuroscience, Bambino Gesu’Hospital Instituto di Ricovero e Cura a Carattere Scientifico (IRCCS), Rome, Italy; 5 Institute of Metabolic Disease, Baylor Research Institute, Dallas, Texas, United States of America; 6 Assistance Publique-Hôpitaux de Paris, Hôpital Armand Trousseau, Service de Neuropédiatrie, Paris, France; 7 INSERM U676, Hopital Robert Debré, Paris, France; 8 Université Pierre et Marie Curie, Paris, France; 9 Soluscience, Faculté de Médecine, Clermont-Ferrand, France; 10 Assistance Publique-Hôpitaux de Paris, Hôpital Robert Debré, Service de Neuropédiatrie et Maladies Métaboliques, Paris, France; 11 Université Paris Diderot, Sorbonne Cité, Paris, France; 12 Centre Hospitalier Universitaire de Clermont-Ferrand, Service de Biochimie Médicale et Biologie Moléculaire, Clermont-Ferrand, France; International Centre for Genetic Engineering and Biotechnology, Italy

## Abstract

Leukodystrophies (LD) are rare inherited disorders that primarily affect the white matter (WM) of the central nervous system. The large heterogeneity of LD results from the diversity of the genetically determined defects that interfere with glial cells functions. Astrocytes have been identified as the primary target of LD with cystic myelin breakdown including those related to mutations in the ubiquitous translation initiation factor eIF2B. EIF2B is involved in global protein synthesis and its regulation under normal and stress conditions. Little is known about how eIF2B mutations have a major effect on WM. We performed a transcriptomic analysis using fibroblasts of 10 eIF2B-mutated patients with a severe phenotype and 10 age matched patients with other types of LD in comparison to control fibroblasts. ANOVA was used to identify genes that were statistically significantly differentially expressed at basal state and after ER-stress. The pattern of differentially expressed genes between basal state and ER-stress did not differ significantly among each of the three conditions. However, 70 genes were specifically differentially expressed in eIF2B-mutated fibroblasts whatever the stress conditions tested compared to controls, 96% being under-expressed. Most of these genes were involved in mRNA regulation and mitochondrial metabolism. The 13 most representative genes, including genes belonging to the Heterogeneous Nuclear Ribonucleoprotein (HNRNP) family, described as regulators of splicing events and stability of mRNA, were dysregulated during the development of eIF2B-mutated brains. *HNRNPH1, F* and *C* mRNA were over-expressed in foetus but under-expressed in children and adult brains. The abnormal regulation of HNRNP expression in the brain of eIF2B-mutated patients was concomitant with splicing dysregulation of the main genes involved in glial maturation such as *PLP1* for oligodendrocytes and *GFAP* in astrocytes. These findings demonstrate a developmental deregulation of splicing events in glial cells that is related to abnormal production of HNRNP, in eIF2B-mutated brains.

## Introduction

Leukodystrophies (LD) represent a heterogeneous group of rare genetic disorders primarily affecting the white matter (WM) of the central nervous system (CNS). The WM is a complex structure composed of a vast number of axons unsheathed by a compact and lipid-rich membrane, the myelin. Beside myelinated axons, WM contains a variety of cells known as glial cells (astrocytes, oligodendrocytes and microglial cells) that play structural, metabolic and trophic roles for myelin and axons [1]. The large heterogeneity of LD results from the diversity of the genetically determined defects that interfere with glial cells functions. A common point in the LD physiopathology is the demyelination and/or development failure (hypomyelination) of CNS myelin from various origins [2]. The hypomyelinating group of LD includes diseases due to defects in myelin production or quality. The main causative gene of hypomyelinating LD controls by means of oligodendrocytes the production of the major brain myelin proteins, the proteolipid proteins (PLP). The demyelinating group of LD includes mainly defects in i) peroxisomal or lysosomal enzymatic activities important for myelin biogenesis and maintenance, or ii) astrocytes functions responsible for progressive cystic myelin breakdown. This last group of vacuolating LD involves mainly genes expressed in astrocytes such as *GFAP* (*Glial Fibrillary Acidic Protein*) and *MLC1* (*Megalencephalic Leukoencephalopathy with subcortical Cysts 1*) or genes ubiquitously expressed as the five *EIF2B1-5* genes encoding the general translation eukaryotic initiation factor 2B (eIF2B).

EIF2B mutations have been initially described in childhood ataxia with central hypomyelination (CACH)/Vanishing white matter (VWM) syndrome characterised in infants by a progressive neurological deterioration exacerbated by episodes of febrile infections or head trauma and a cerebrospinal fluid-like signal intensity of the WM on brain magnetic resonance imaging [3], [4]. EIF2B mutations have been subsequently observed in a wide clinical spectrum from congenital rapidly lethal forms to slowly progressive adult forms associated in some cases with ovarian failure [5]–[9]. A correlation between age at disease onset and disease severity has been established: disease onset <2, from 2 to 5 or >5 years are associated respectively to severe, classical or mild phenotypes [10].

eIF2B is involved in the translation initiation regulation particularly under cellular stress by activating the eIF2 complex thanks to its nucleotide guanine exchange factor (GEF) activity. A correlation between the eIF2B GEF activity and the disease severity has been established particularly in the most severely affected infantile group [11], [12]. A hyper-activation of the endoplasmic reticulum (ER)-stress response mediated by the activating transcription factor 4 (ATF4) has been observed in rat oligodendroglial-derived cells expressing mutated human *EIF2B5* gene [13] as well as in primary fibroblasts [14] and cerebral WM from eIF2B-mutated patients [15], [16] but not in lymphocytes and lymphoblasts from eIF2B-mutated patients [17] suggesting cell-specific response to this genetic defect.

The neuropathological features of eIF2B related disorders show an increased oligodendrocytic density [18], [19] and a reduced number of dystrophic astrocytes due to their abnormal maturation [20], suggesting an abnormal glial cell maturation in the WM susceptibility to eIF2B mutations. Moreover, mild transitory abnormal myelination has been reported in mice homozygous for a mutant *Eif2b5* allele (*Eif2b5* ^R132H/R132H^ mice) with an early defect of glial cells proliferation and maturation [21].

In order to identify genes and metabolic pathways specifically involved in eIF2B-related disorders, we performed a differential transcriptomic analysis using patients’ fibroblasts with or without ER stress conditions. Fibroblasts of eIF2B-mutated patients with a severe, early infantile form, were compared to age matched fibroblasts of controls as well as patients with other types of LD (OL-patients). We described here for the first time genes involved in the mRNA splicing machinery as actors in the abnormal maturation of glial cells observed in the eIF2B-mutated patient brains.

## Results

### 70 Genes Specifically and Differentially Expressed in eIF2B-mutated Fibroblasts

In order to identify genes specifically involved in eIF2B-related disorders, we performed a transcriptomic analysis comparing fibroblasts of 10 eIF2B-mutated patients with 10 of OL-patients and with to 10 age matched control fibroblasts (Table 1). ANOVA was used to identify genes that were statistically significantly differentially expressed between eIF2B-mutated and OL-cells at basal state and after ER-stress by thapsigargin treatment. Thapsigargin is a blocker of the ER calcium pump. The pattern of differentially expressed genes between basal state and thapsigargin treatment in the eIF2B-mutated, OL and control cells did not differ significantly. These results suggest that ER-stress does not enhance the effect of eIF2B mutations and is probably not directly involved in the specific expression profile of eIF2B-related disorders in cultured skin fibroblasts.

**Table 1:** Characteristics of subjects and fibroblasts used in our differential transcriptomic analysis.

Affected patient number	Type ofLD	Age at disease onset	Disease duration*	Mutated gene	Molecular or biochemical defect	Gender	Age skin biopsy	Fb passages	Control subject number	Gender	Age at skin biopsy	Fb passages
291-1	ERD	2.3 y	2.7 y	*EIF2B5*	c.338G>A/c.1160A>G	F	9 y	5	1530-1	F	9 y	7
375-1	ERD	2.6 y	3 y	*EIF2B5*	c.338G>A/c.1948G>A	F	2,7 y	4	1545-1	F	16 m	5
393-1	ERD	0.9 y	0.7 y	*EIF2B5*	c.925G>C/c.925G>C	F	13 m	3	1540-1	F	15 m	8
393-2	ERD	0.8 y	0.8 y	*EIF2B5*	c.925G>C/c.925G>C	F	11 m	4	1541-1	F	9 m	6
431-1	ERD	1,5 y	3.5 y	*EIF2B5*	c.338G>A/c.1884G>A	F	3,6 y	4	1178-1	F	5 y	10
432-2	ERD	1.5 y	2.5 y	*EIF2B5*	c.271A>G/c.1015C>T	M	3,5 y	7	1596-1	M	3 y	8
590-2	ERD	1 y	3.5 y	*EIF2B5*	c.1028A>G/c.1153A>G	F	3 y	3	1178-2	F	3 y	8
894-1	ERD	0.6 y	10 d	*EIF2B5*	c.584G>A/c.584G>A	M	7 m	3	1539-1	M	8 m	8
894-2	ERD	0.4 y	10 d	*EIF2B5*	c.584G>A/c.584G>A	F	5 m	3	1538-1	F	6 m	11
1036-1	ERD	0.8 y	6 m	*EIF2B5*	c.967C>T/c.1280C>T	M	12 m	3	1537-1	M	10 m	9
351-1	AD	1 y	NA	*GFAP*	c.729C>T	F	7 y	4	1178-3	F	8 y	10
672-1	AD	6 m	NA	*GFAP*	c.243A>T	F	6,6 y	5	1594-1	F	7 y	6
1303-1	AD	NA	NA	*GFAP*	c.249C>T	F	3,5 y	4	1178-2	F	3 y	8
256-1	MLC	3 y	NA	*MLC1*	c.249G>T/c.IVS5+6T>G	M	15 y	5	1178-4	M	17 y	10
773-1	MLC	3,5 y	NA	*MLC1*	c.135insC/c.135insC	F	4,7 y	3	1178-1	F	5 y	10
1143-1	SLS	2 m	NA	*ALDH3A2*	decreased FALDH activity	F	4 y	6	1600-1	F	4 y	6
1179-1	SLS	At birth	NA	*ALDH3A2*	decreased FALDH activity	M	3 y	9	1596-1	M	3 y	10
771-1	KB	3.5 m	NA	*GALC*	decreased b-gal activity	F	8 m	3	1539-1	F	8 m	9
167-1	PMD	1 m	NA	*PLP1*	*PLP1* duplication	M	4 y	7	1178-5	M	5 y	7
767-1	PMD	At birth	NA	*PLP1*	c.454-1G>A/c.454-1G>A	M	2 y	4	1598-1	M	2 y	5

LD: leukodystrophy, ERD: eIF2B-related disorder, AD: Alexander disease, MLC: Cystic Megalencephalopathy, SLS: Sjögren-Larsson syndrome, KB: Krabbe disease, PMD: Pelizaeus-Merzbacher disease, y: year, m: months, d: day, b-gal: beta-galactosidase, F: female, M: male, Fb: fibroblasts, NA: not available. *disease duration in year, corresponding to the time between age at disease onset and death or confinement to bed with loss of neurodevelopment abilities and need of constant assistance.

Two hundred fifty three genes were differentially expressed (FDR≤0.12%) between the eIF2B-mutated fibroblasts and control fibroblasts whatever the stress conditions tested. Among these 253 genes, 70 genes were specifically differentially expressed in eIF2B-mutated cells when compared with OL fibroblasts, and with significant expression rate, considered as significant mean ratios eIF2B-mutated/mean control values ≤0.9 or ≥1.05 (Table S1). Sixty-seven of the 70 genes (96%) were under-expressed whereas only three genes were weakly over-expressed (range over-expression rate 1.06 to 1.07). These under-expressed genes are involved in (a) transcription or mRNA stabilization and splicing (25%), (b) mitochondria metabolism (15%), (c) development (12%), (d) cell cycle (10%), (e) DNA compaction or repair (8%), (f) cytoskeleton (5%), (g) protein synthesis (4%), and (h) other metabolic pathways (21%) (Table S1).

### RNA Processing Deregulation in eIF2B-mutated Fibroblasts

The 70 genes were analysed using the BBSPE function of the Genomatix software, in order to select biological processes mostly and specifically involved in eIF2B-related disorders. We found that the highest number of genes were all linked to RNA process: mRNA metabolic process (z-score: 10.08), mRNA processing (z-scores: 9.74 and 8.91) and mRNA splicing (z-scores: 9.54 to 8.03). This group statistically diverges form the other biological processes with a z-score difference of 1. As an example, 8.6% from these 70 deregulated genes are HNRNP genes.

### Expression Deregulation of Several Genes in eIF2B-mutated Fibroblasts Confirmed by mRNA Quantification

We selected 10 under-expressed genes for microarray data validation according to their putative link with the physiopathology of eIF2B-related disorders (Table 2). Among the group of 70 differentially expressed genes described above, we first chose three genes involved in transcription or mRNA stabilization and splicing: *HNRNPH1, HNRNPL* and *HNRNPC* due to the tight regulation of the alternative splicing of the major myelin protein mRNA during myelin formation and maintenance [22]–[25]. We added also the *HNRNPF* gene to the analysis, yet not selected using our statistical approach (FDR>0.12 for this gene), due to the particular role of hnRNPH1 and hnRNPF controlling the *PLP1/DM20* mRNA alternative splicing described recently in oligodendrocytes [26]. We also selected among our group of 70 differentially expressed genes five genes related to mitochondria structure and metabolism, *MRPS26 (Mitochondrial Ribosomal Protein S26), MRPL28 (Mitochondrial Ribosomal Protein L28), HCCS (Holocytochrome c Synthase), VDAC3 (Voltage-Dependent Anion-selective Channel protein 3)* and *KIF5B (Kinesin Family member 5B)* due to the potential key role of mitochondria in WM homeostasis as suggested by the involvement of mitochondrial defects in various neurodegenerative disorders [27] including KIF5A in one inherited progressive primary motor axonopathy (SPG10) [28]. One gene related to follicle development and spermatogenesis, *DIAPH3 (Diaphanous Homolog 3)*, the human homolog of the drosophila *Diaphanous* gene [29], was also selected due to the frequent ovarian dysfunction observed in eIF2B-related disorders.

**Table 2:** List of the genes selected for QRT-PCR analysis.

Gene name	Description	Taqman Gene Expression Assay reference
*HNRNPF*	Heterogeneous nuclear ribonucleoprotein F	Hs01014497_ml
*DIAPH3*	Diaphanous homolog 3	Hs01107326_ml
*VDAC3*	Voltage-dependent anion-selective channel protein 3	Hs01091534_ml
*HNRPL*	Heterogeneous nuclear ribonucleoprotein L	Hs00704850_gl
*MRPL28*	Mitochondrial ribosomal protein 28 L	Hs00371771_ml
*HNRPH1*	Heterogeneous nuclear ribonucleoprotein H	Hs01033845_gl
*HCCS*	Holocytochrome C synthase	Hs00403938_ml
*HNRPC*	Heterogeneous nuclear ribonucleoprotein C	Hs01028912_ml
*MRPS26*	Mitochondrial ribosomal protein 26 S	Hs00258287_ml
*KIF5B*	Kinesin family member 5 B	Hs00189659_ml
*HNRNPU*	Heterogeneous nuclear ribonucleoprotein U	Hs00244919_m1
*HNRNPD*	Heterogeneous nuclear ribonucleoprotein D	Hs00606052_m1
*HNRNPR*	Heterogeneous nuclear ribonucleoprotein R	Hs00195167_m1
*PLP*	Proteolipid protein 1	Mm00456892_m1
*PLP/DM20*	Proteolipid protein 1	Mm00456894_m1

Gene names, descriptions and Taqman Gene Expression Assay reference used for QRT-PCR quantitation are mentioned.

We quantified the mRNA level of the 10 selected genes by QRT-PCR on the same RNA samples used for microarray analysis: 10 eIF2B-mutated patient/control fibroblasts couples and 10 OL-patient/control fibroblasts couples in stressed and non stressed conditions. For each gene, the determination correlation coefficient (R^2^) between microarray normalized log ratios patient/control and QRT-PCR delta Ct (patient-control) have been calculated (Table 3). Among the ten eIF2B-mutated patient/control couples, lack of correlation between QRT-PCR and microarray data for 8 of the selected genes (except *HNRNPH1* and *MRPS26* genes) was found for two couples (393-2/1541-1 and 431-1/1178-1, data not shown); therefore these couples were subsequently excluded for the global correlation analysis. For six genes, the correlation coefficients were significantly higher (range of p-value 0 to 0.030) in the 8/10 eIF2B-mutated patient/control fibroblasts couples than in the 10 OL-patient/control fibroblasts couples (Table 3). The difference between eIF2B-mutated/control and OL-patient/control couples was high but not significant for the *HCCS* and *HNRNPC* genes (respective p-value  = 0.152 and 0.144). No significant difference was observed for the *KIF5B* and *MRPS26* genes (respective p-value  = 0.876 and 0.736). These results confirm the specificity of the quantitative differential expression of 6/10 selected genes in the eIF2B-mutated patient’s fibroblasts.

**Table 3:** Recapitulative data of microarray and QRT-PCR experiments.

Gene name	FDR (%) eIF2B-pathy	FDR (%) OL	Microarray expression rate eIF2B-pathy	Microarray expression rate OL	QRT-PCR expression rate eIF2B-pathy	QRT-PCR expression rate OL	R^2^ eIF2B-pathy	R^2^ OL	P-value
***HNRNPF***	1.1	6.93	0.88±0.15	1.16±0.42	0.76±0.15	1.23±0.49	0.888	0.05	0.000
***DIAPH3***	0.12	77.39	0.84±0.24	0.97±0.28	0.60±0.13	1.01±0.36	0.711	0.203	0.009
***VDAC3***	0.05	93.98	0.82±0.19	1.02±0.43	0.77±0.17	1.14±0.30	0.594	0.07	0.008
***HNRPL***	0	82.58	0.77±0.22	1.00±0.21	0.80±0.18	1.06±0.26	0.555	0.033	0.000
***MRPL28***	0.07	61.09	0.86±0.14	1.01±0.23	0.76±0.11	1.13±0.19	0.497	0.004	0.004
***HNRPH1***	0	5.02	0.66±0.15	1.01±0.38	0.75±0.13	1.09±0.23	0.479	0.051	0.030
***HCCS***	0.01	12.91	0.84±0.12	0.93±0.41	1.01±0.17	1.03±0.28	0.337	0.062	0.152
***HNRPC***	0.04	48.18	0.78±0.23	1.01±0.24	0.83±0.20	1.11±0.27	0.117	0.004	0.144
***MRPS26***	0.07	15.24	0.86±0.13	1.09±0.32	0.94±0.21	1.22±0.54	0.016	0.001	0.736
***KIF5B***	0.01	40.18	0.86±0.11	0.94±0.43	0.81±0.16	0.96±0.25	0.01	0.003	0.876

Data obtained in eIF2B-mutated and OL fibroblasts for the 10 selected genes and correlation analysis between microarray and QRT-PCR data. Expression rate of microarray were calculated using the formula Mean ((Corrected G log value)i/(Corrected G log value)j) were (Corrected G log value) were calculated as explained in material and methods. i correspond to a patient’s fibroblasts and j to a control’s fibroblasts. The (Corrected G log value) between the ethanol and thapsigargin conditions were not different for the 10 selected genes which allowed us to mean them. Expression rate of QRT-PCR were calculated using the formula Mean (2^∧^(delta (patient)- delta (control))) where delta (patient) correspond to (Ct gene *x* − Ct B2M) for one patient and delta (control) to (Ct gene *x* − Ct B2M) for one control. The 2^∧^(delta (patient)- delta (control)) between the ethanol and thapsigargin conditions were not different for the 10 selected genes which allowed us to mean them. The two correlation coefficients are transformed with the Fisher Z-transform Zf  = 1/2 * ln((1+R)/(1-R) ) and the difference z  =  (Zf1 - Zf2)/SQRT(1/(N1−3)+1/(N2−3) ) is approximately Standard Normal distributed. Then the p-value of the difference has been calculated. For six genes, the determination coefficient (R^2^) is significantly higher in 8/10 eIF2B-mutated patient/control fibroblasts couples than in the 10 OL patient/control fibroblasts couples. The difference between eIF2B-mutated/control and OL patient/control couples is high but not significant for the *HCCS* and *HNRNPC* genes and no significant differences is observed for the *KIF5B* and *MRPS26* genes. FDR: False Discovery Rate value.

### Expression of Several Genes Deregulated During the Development of eIF2B-mutated Brains

**Figure 1:**
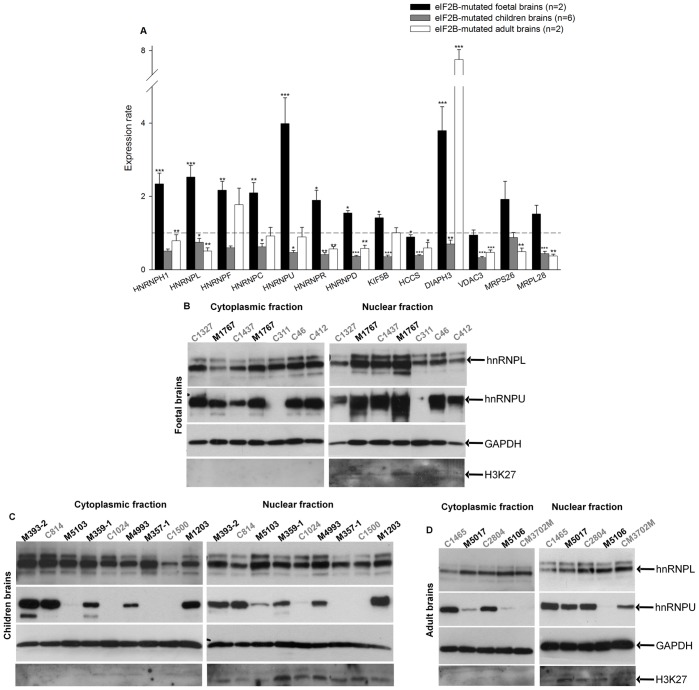
Expression rates of the 13 selected mRNA and two hnRNP proteins in eIF2B-mutated fœtal and patients brains. A. RNA was isolated from brains and analyzed by quantitative RT-PCR for expression of the 13 selected genes. Expression rates were calculated using the formula Mean (2^∧^-(delta (patient)- delta (control))) where delta (patient) correspond to (Ct gene *x* − Ct *B2M*) for one patient and delta (control) to (Ct gene *x* − Ct *B2M*) for the corresponding control. Errors bars represent standard error (s.e.m.) (*P<0.05, **P<0.01, ***P<0.001). **B–D.** To assess the expression of hnRNPL and hnRNPU proteins in the brain autopsy samples, a nuclear and cytoplasmic differential extraction were performed followed by western blot analysis using antibodies specific for hnRNPL and U. GAPDH served as loading control. H3K27 served as a nuclear control. Are represented in **B** the fetuses samples, in **C** the children samples and in **D** the adults samples. C: control, M: eIF2B-mutated.

We assayed the mRNA level of the 10 selected genes by QRT-PCR in the brain autopsy samples of two eIF2B-mutated foetuses, seven children and two adults age matched with controls (Tables 4 and 5). We also included in this study three additional HNRNP genes (*HNRNPU*, *HNRNPD* and *HNRNPR*), that were present in the 70 initial genes, due to the high representation of the HNRNP genes (8.6%) among the deregulated genes. An over-expression was globally observed in eIF2B-mutated foetal brains compared to controls except for *HCCS* and *VDAC3* whose mRNA expression did not vary (Figure 1A). Nevertheless, a lower mRNA expression was globally observed in the eIF2B-mutated children and adult brain samples compared to the age-matched control brains, excluding *DIAPH3* that was under-expressed in the eIF2B-mutated children brains but over-expressed in the adult brains (Figure 1A). These results demonstrated that genes specifically under-expressed in eIF2B-mutated fibroblasts of children, in comparison to controls and to OL-patients, are also globally under-expressed in eIF2B-mutated children brains. Interestingly, these genes are over-expressed in eIF2B-mutated foetal brains suggesting a developmental deregulation.

**Table 4:** Characteristics of brain samples used for microarray data validation on QRT-PCR (qPCR) and/or western blot (WB) analysis.

Patient number		Age at autopsy	Gender	Mutated gene	cDNA mutation	Usage
**1327**		Fœtus 15w	F	WT	WT	qPCR, WB
**1437**		Fœtus 15w	M	WT	WT	qPCR, WB
**412**		Fœtus 19w	M	WT	WT	qPCR, WB
**46**		Fœtus 19w	F	WT	WT	qPCR, WB
**917**		Fœtus 19w	M	WT	WT	qPCR
**311**		Fœtus 24w	F	WT	WT	qPCR, WB
**393**		Fœtus 14w	M	*EIF2B5*	p.Val309Leu/p.Val309Leu	qPCR
**1767**		Fœtus 16w	F	*EIF2B5*	p.Ala239Pro/p.Ala239Pro	qPCR,WB
**814**		Child 16m	M	WT	WT	qPCR, WB
**1500**		Child 7y	M	WT	WT	qPCR, WB
**1024**		Child 14y	M	WT	WT	qPCR, WB
**393-2**		Child 13m	F	*EIF2B5*	p.Val309Leu/p.Val309Leu	qPCR, WB
**5103**		Child 16m	M	*EIF2B*	NA	qPCR, WB
**357-1**		Child 4y	M	*EIF2B5*	p.Arg136Cys/p.Arg339Trp	WB
**359-1**		Child 10y	M	*EIF2B4*	p.Pro243Leu/p.Pro243Leu	qPCR, WB
**NA**		Child	NA	*EIF2B*	NA	qPCR
**1203**		Child 7y	M	EIF2B5	p.Gly386Val/p.Arg113His	qPCR, WB
**4993**		Child 12y	M	*EIF2B*	NA	qPCR, WB
**1465**		Adult 17y	M	WT	WT	qPCR, WB
**M3702M**	Adult 19y		M	WT	WT	qPCR, WB
**2804**		Adult 26y	M	WT	WT	qPCR, WB
**5017**		Adult 24y	M	*EIF2B*	NA	qPCR, WB
**5106**		Adult 39y	F	*EIF2B5*	p.Pro454Ser/p.Arg113His	qPCR, WB

y: years, m: months, w: weeks, NA: not available, M: male, F: female, WT: wild type.

We then focused our analysis on the protein levels of HNRNP, and particularly on HNRNPL and U by testing one eIF2B-mutated foetus brain (only one brain was available for protein extractions), six eIF2B-mutated children brains and two eIF2B-mutated adult brains in comparison to associated controls. As shown in Figure 1B, the relative amounts of these proteins seem to increase in the nuclear fraction of the eIF2B-mutated foetal brain, consistently with the previous results on transcripts found in two affected foetus brains. Moreover, we can observe an over-expression of HNRNPL in the nuclear fraction of the eIF2B-mutated foetus brain compared to cytoplasmic fraction, which is totally inverted in the control brains. Nevertheless, the protein analysis of only one foetal brain did not allow us to demonstrate a statistically significant deregulation (Figure 1B). Protein analysis of the children (Figure 1C) and adult brains (Figure 1D) using western blotting showed variability in HNRNL and U expressions that did not allow us to demonstrate a statistically significant deregulation between control and eIF2B-mutated brains at the protein level.

### Abnormal Splice Regulation of PLP1 and GFAP in Brains from eIF2B-mutated Patients

**Figure 2:**
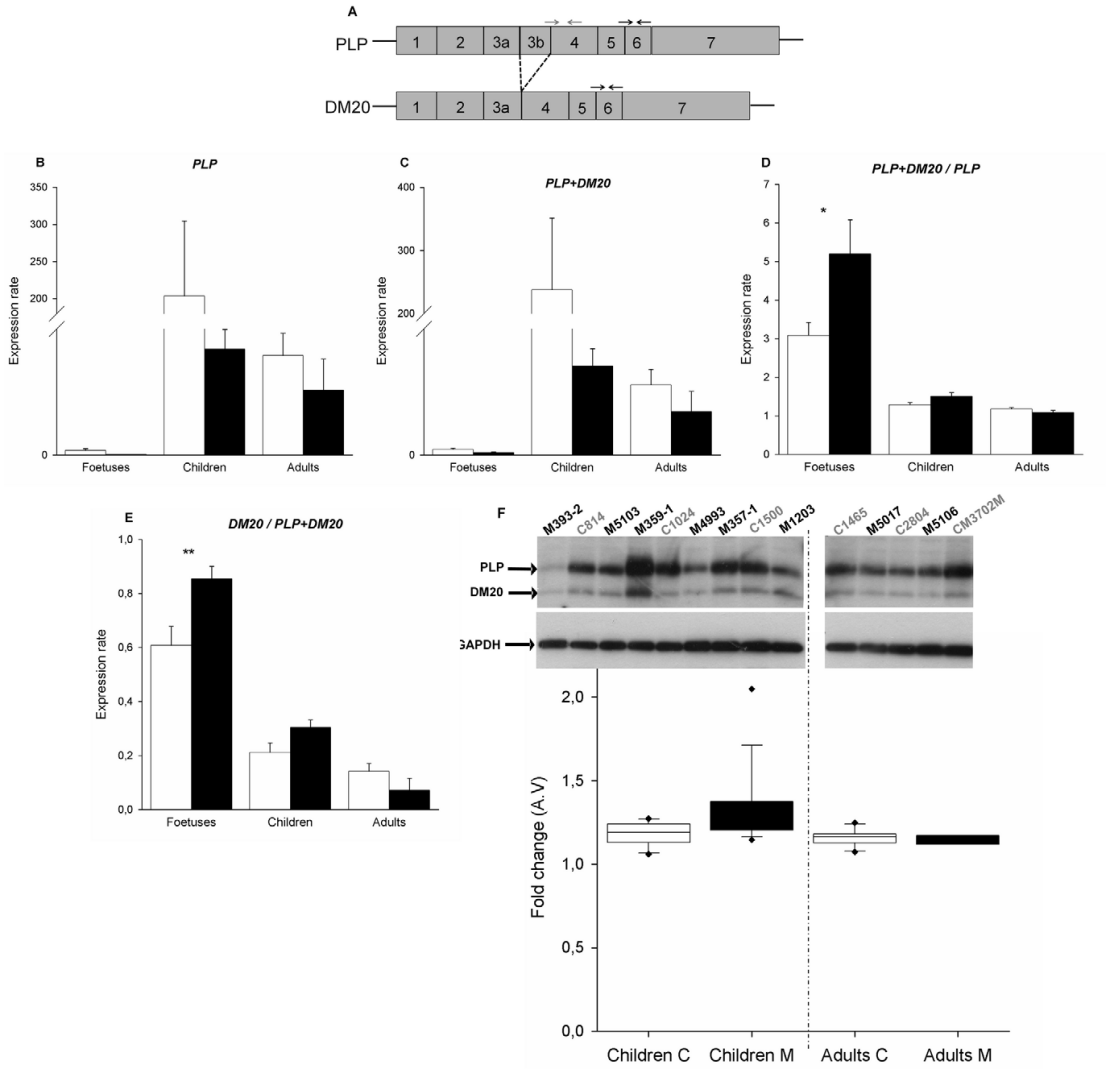
Study of the splice *PLP/DM20* in eIF2B-mutated patients brains. A. Transcripts of human *PLP1* splice variants. The boxes represent the exons that are transcribed for the different isoforms. The black and grey arrows represent respectively common primers between *PLP* and *DM20* (forward primer between the exons 5 and 6, reverse primer at the end of the exon 6), and specific primers for *PLP* (forward primer between the exons 3b and 4, reverse primer in the exon 4). **B, C.** RNA was isolated from control brains (white bars) *vs* eIF2B-mutated patient brains (black bars) and analyzed by quantitative RT-PCR for expression of *PLP* and *PLP+DM20*. Expression rates were calculated using the formula Mean (2^∧^-(Ct gene *x* − Ct *B2M*)) and ratio *PLP+DM20/PLP* was performed (**D**). **E.** Extrapolation of the *DM20/PLP+DM20* ratio calculating *DM20* alone by the formula (*PLP+DM20*)-*PLP*. Errors bars represent standard error (s.e.m.) (*P<0.05,**P<0.01, ***P<0.001).**F.** Proteins were extracted from controls and eIF2B-mutated patient brains and western blot analysis was performed on the cytoplasmic fractions using antibodies specific for PLP and DM20. GAPDH served as loading control. C: control, M: eIF2B-mutated. The images were quantified using ImageQuant TL software and the box plots below show the relative expression of PLP+DM20/PLP (corrected for GAPDH levels) in control and patients brains. A.V.: arbitrary value.

We next wanted to know if the deregulation observed in the expression of genes implicated in the mRNA stabilization and splicing (e.g. *HNRNP*) has functional consequences. For this purpose, we first studied the *PLP/DM20* mRNA alternative splicing, known to be regulated during oligodendrocytes maturation by hnRNPH1 and hnRNPF: reduction in *HNRNPH1* and *F* expression in differentiated mouse oligodendrocytes correlates temporally with increased Plp/Dm20 ratio [26]. *DM20* results from an alternative splicing of the *PLP1* gene resulting in exclusion of part of the exon 3 (Figure 2A). To gain insight into the splice *PLP/DM20* in the eIF2B-mutated patients’ brains, we quantified by QRT-PCR the isoform *PLP* alone and *PLP+DM20* in the brain autopsy samples previously used for HNRNP analysis (Table 3). As expected, *PLP* and *PLP+DM20* transcripts were undetectable or very low in the control foetal brains, and increasing in the children/adult brains consecutively to the myelination (Figure 2B, C). *PLP* and *PLP+DM20* transcripts remained under-expressed in the eIF2B-mutated brains compared to controls, in correlation with the hypomyelination observed in eIF2B-related disorders (Figure 2B, C). However, the *PLP+DM20/PLP* ratio was significantly increased in eIF2B-mutated foetal brains compared to controls (Figure 2D). As a result, the extrapolation of the calculated DM20 expression alone (*PLP+DM20*)-*PLP*, and the representation of DM20/PLP+DM20 showed an increased ratio in eIF2B-mutated foetal brains compared to controls (Figure 2E). These results demonstrated an abnormal upregulation of the DM20 isoform during foetal life in the affected brains.

The protein levels of PLP and DM20 and the quantification of the ratio PLP+DM20/PLP showed no differences between the control and patient brains at the children/adult stage, correlating with the QRT-PCR results (Figure 2F). PLP protein was undetectable in the foetal brains. These results showed a defect in the regulation of the *PLP/DM20* mRNA splice or stability at an early stage of brain development.

**Figure 3:**
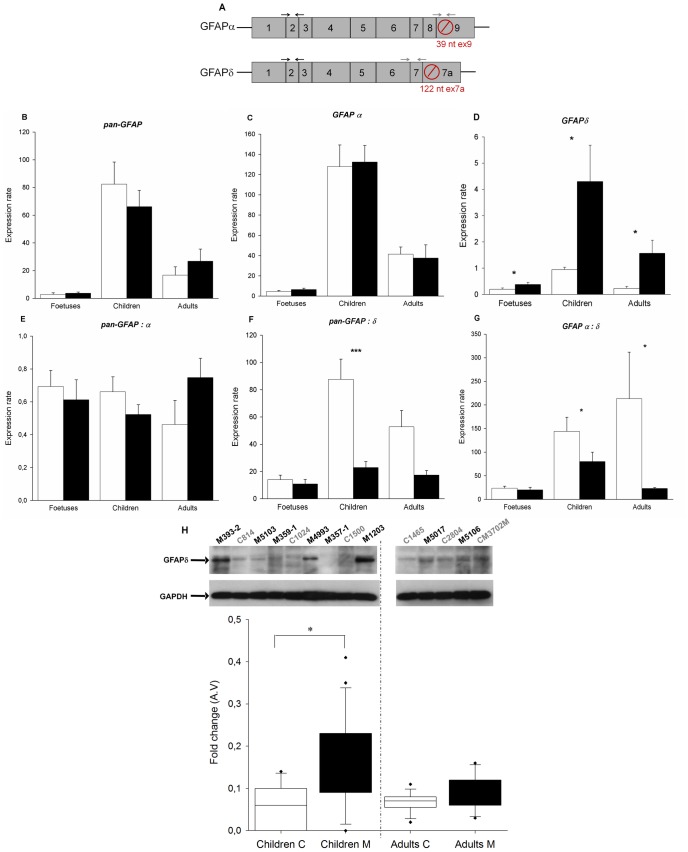
Study of the splice *GFAP* in eIF2B-mutated patients brains. A. Transcripts of human *GFAP* splice variants. The boxes represent the exons that are transcribed for the different isoforms. The termination codon, indicated by a blocked round circle, is located in exon 9 for *GFAPalpha*, and in exon 7a for *GFAPdelta*. The black and grey arrows represent respectively common primers between *GFAPalpha* and *GFAPdelt*α (forward primer between the exons 1 and 2, reverse primer between exons 2 and 3), and specific primers for each isoforms (forward primer between the exons 8 and 9, reverse primer in the exon 9 for the *GFAPalph*α isoform, forward primer in the exon 6, reverse primer between the exons 7 and 7a for the *GFAPdelt*α isoform). **B-D.** RNA was isolated from control brains (white bars) *versus* eIF2B-mutated patient brains (black bars) and analyzed by quantitative RT-PCR for expression of *pan-GFAP, GFAPalpha* and *GFAPdelta*. Expression rates were calculated using the formula Mean (2^∧^-(Ct gene *x* − Ct *B2M*)). Ratios were performed between *pan-GFAP* and *GFAPalpha (E) pan-GFAP* and *GFAPdelt*α (**F**), *GFAPalpha* and *GFAPdelt*α (**G**). Errors bars represent standard error (s.e.m.) (*P<0.05,**P<0.01,***P<0.001). **H.** Proteins were extracted from controls and eIF2B-mutated patient brains and western blot analysis was performed on the cytoplasmic fractions using antibodies specific for GFAP delta. GAPDH served as loading control. C: control, M: eIF2B-mutated. The images were quantified using ImageQuant TL software and the box plots below show the relative expression of PLP+DM20/PLP (corrected for GAPDH levels) in control and patients brains. A.V.: arbitrary value.

Similarly, we quantified the astrocytic differentially expressed isoforms of *GFAP, GFAPalph*α and *GFAPdelt*α (Figure 3A) in the same brain autopsy samples (Table 3). *Pan-GFAP* mRNA and the two isoforms *GFAPalph*α and *GFAPdelt*α were amplified by QRT-PCR. The normal expression of the *Pan-GFAP* mRNA and its isoform alpha and delta is low at the foetal stage, reaches a peak during childhood and decreases by adult age (Figure 3B, C, D). *Pan-GFAP* and *GFAPalpha* mRNA are not differentially expressed between the control and eIF2B-mutated samples at the 3 stages of development (Figure 3B, C). As a consequence, we observed an equivalent ratio *pan-GFAP*/*GFAPalpha* mRNA in the control and eIF2B-mutated patient brains (Figure 3E). However, *GFAPdelta* mRNA was significantly over-expressed in the eIF2B-mutated patient brains at every development stages (Figure 3D). The ratios *pan-GFAP/GFAPdelta* and *GFAPalpha/GFAPdelta* increased in the eIF2B-mutated children/adult brains compared to controls but not in the eIF2B-mutated foetus brains (Figure 3F, G). A specific upregulation of the *GFAPdelta* mRNA isoform was 4.5 and 7 times higher respectively in eIF2B-mutated children and adult brains. Western blotting analysis confirmed that GFAPdelta was easier to detect in the eIF2B-mutated post-natal brains than in controls with a statistically significant difference in children samples (Figure 3H, antibody GFAPalpha not available). Similarly to PLP, GFAP proteins were undetectable in the foetal brains. These results demonstrate an abnormal splicing or unstable regulation of *GFAP* in the eIF2B-mutated patients brains that is more pronounced at the post-natal stage than in foetal stage.

## Discussion

### Differential Transcriptomic Analysis in Rare Neurological Disorders needs Comparison by Variance Analysis of Related Groups of Disease

In rare inherited neurological disorders, DNA microarray experiments are directed to detect differential gene expression in order to identify genes or metabolic pathways important for diagnostic, prognostic or therapeutic approaches. The novelty of our study has been to compare transcriptomic profiles of fibroblasts from eIF2B-mutated patients with fibroblasts of OL-patients in addition to fibroblasts from control subjects. Moreover, we consider that comparing the fold change results is a misleading information offered by microarray experiments [30], [31]. We used analysis of variance with the GeneANOVA software [32] to compare the data taking into account different factors such as disease status, couple number and ER-stress treatment between the different groups of fibroblasts: eIF2B-mutated, OL and controls. This type of statistical analysis allowed us to maximize the use of available data (relative transcription level of the corresponding gene and systematic bias due to the protocol such as labelling efficacy).

### ER-stress Activation is not Specific to eIF2B-related Disorders

A hyper-activation of ER-stress response has been reported in fibroblasts from eIF2B-mutated patients [14], or rat oligodendroglial-derived cells expressing mutated human *EIF2B5* gene [13]. An UPR (unfolded protein response) hyper-activation has also been described in brains from eIF2B-mutated patients compared to control individuals [15] and to a single PMD case [16]. Our transcriptomic analysis showed that ER-stress induced by thapsigargin did not help to differentiate gene expression profile of eIF2B-mutated fibroblasts from leucodystrophic fibroblasts of other causes. We explain this discrepancy as our 10 eIF2B-mutated patients expressed a homogenous severe phenotype and were compared for the first time to a group of infantile severe forms of leukodystrophic patients from various other causes including patients with other type of vacuolating LD. This suggests that the ER-stress response observed in eIF2B-mutated cells has no specificity despite the role of the eIF2B complex in translation regulation after stresses. Stress, classically reported as an onset trigger or aggravating factor in eIF2B-related disorders [9], [33], also worsened symptoms in other groups of WM disorders particularly in other types of vacuolating LD. The functions of the ER in proper folding of proteins, lipids biosynthesis and storage of calcium could explain the susceptibility of the WM to ER stress, particularly during the myelination process that requires rapid synthesis of large amount of proteins and lipids [34].

### Specific Genes Involved in eIF2B-related Disorders are Under-expressed and Belong Mainly to mRNA Regulation and Mitochondrial Functions

Our transcriptomic analysis identified 70 genes that were specifically differentially expressed in eIF2B-related disorders and belonging to three main biological processes.

*The first group gathers 25% of the selected genes which are involved in transcription or mRNA stabilization and splicing*, with a high z-score for the associated biological processes, suggesting a major disturbance of mRNA regulation. We identified six genes belonging to the *HNRNP* family (*HNRNPH1/C/D/L/U/F* and *R*) and two *MBNL* genes (*MBNL1* and *MBNL2*: Muscle blind like 1 and 2) which were involved in mRNA splicing or mRNA stability and transport suggesting that splicing and stabilization of a large panel of mRNA can be affected in the eIF2B-related disorders. Alternative splicing, widely used to generate protein diversity and to control gene expression, is highly abundant in brain relative to other tissues [35], [36]. It may generate cell-specific combination of protein isoforms that define the functional properties of the cells and underline complex processes such as synaptic adhesion and CNS plasticity [37]. Most of the main specific myelin proteins are submitted to alternative splicing, such as PLP/DM20 [24], [25], MAG (Myelin-Associated Protein) [22] or MBP (Myelin Basic Protein) [38] and the production of the different isoforms during myelination have to be tightly regulated. Indeed, it has been demonstrated that the hnrnpH1 and F proteins control the alternative splicing of the *Plp/Dm20* mRNA in a mouse oligodendroglial cell line [26]. The knock-down of these hnRNP proteins by siRNA modified the *Plp/Dm20* mRNA ratio by increasing the production of the *Plp* mRNA isoform. Moreover, a recent study demonstrated that over-expression of *HNRNPC2* in a human neuroblastoma cell line up-regulated the message level of *MBP* [39]. The identification of an increase of such a number of *HNRNP* genes in foetal brains together with their decrease in children and adult brains suggests that eIF2B mutations may have a major effect on myelination timing and myelin maintenance. These results are in agreement with the recent microarray analysis performed in *Eif2b5*-mutated mice brains where authors described a down-regulation of oligodendrocyte-specific genes at specific time point during the development [40]. Analysis of the published microarray results of *Eif2b5*-mutated mice brains showed a decrease in Hnrnp genes (e.g. *HnrnpH1*) at postnatal day 18 and 21 (P18 and P21), corresponding to the peak period of myelin formation. Here we identified the first link between eIF2B mutations and a potential myelin defect due to *HNRNPH1* mRNA, a key regulator of the major myelin protein PLP/DM20 splicing and the dynamic of the myelin production during the development. It has also been demonstrated that several HNRNP shuttle between the nucleus and the cytoplasm, transporting mature mRNA for translation or regulating mRNA stability [41]. So, the differential localization of HNRNPL and U in the foetal brains between controls and eIF2B-mutated patients yield to the hypothesis of a functional consequence on target genes and processes of these hnRNP.

### The Second Group of Selected Genes is Related to Mitochondria Metabolism and Transport

Interestingly, five genes encoding mitochondrial ribosomal subunits (*MRPS26*, *MRPL28, MRPL38, MRPL16* and *MRPS9*) were under-expressed. This suggests that protein synthesis in mitochondria is probably also affected by eIF2B mutations. The identification of some genes involved in the electron transport pathway (*HCCS*) or component of the mitochondrial membrane like *VDAC3*, mitochondrial respiratory chain (*SDHD*) and in mitochondrial transport (*KIF5B*), suggests that the global metabolism of mitochondria is affected in eIF2B-related disorders. In CNS, mitochondria are notably important for Ca^2+^ signalling in oligodendrocyte precursor cells which seems to be critical during their migration, proliferation and differentiation [42]. Mitochondria are also essential for neuronal activity [43] and defects in their functions have been involved in various neurodegenerative disorders [27], such as SPG10, due to mutations in the neuron-specific *KIF5A* gene. Moreover, a study showed that Kif1b is required for the localization of *mbp* mRNA to processes of myelinating oligodendrocytes in zebrafish [44].

#### Another interesting gene differentially expressed in eIF2B-mutated patient fibroblasts and brains is the DIAPH

The human homolog of the Drosophila *Diaphanous* gene, involved in cytokinesis [29]. Disruption of the *Diaphanous* gene in Drosophila is responsible for spermatids degeneration and abnormal follicle cells development leading to male and female infertility. Moreover, another human gene belonging to the *Diaphanous* family named *DIA* is disrupted in a patient with premature ovarian failure [45]. DIAPH3 has been recently involved in cell migration, axon guidance and neuritogenesis [46]. We can therefore hypothesize that it may be also involved in folliculogenesis, and would explain the ovarian failure sometimes observed in mild cases of eIF2B-related disorders (ovarioleukodystrophy) [7].

The differences found in genes expression between foetal and children/adult eIF2B-mutated brains suggest that eIF2B mutations have different effects depending on the developmental stage. Nine of the 13 genes tested are significantly over-expressed in foetal brains but 10 and 8 are under-expressed in children and adult brains respectively (Figure 1), suggesting that eIF2B mutations have specific deleterious effects during embryonic life. Our results coupled to the various congenital abnormalities observed in the “congenital” forms of eIF2B-pathies (glaucoma, cataract, dysmorphic features [45]) suggest a larger effect on the programmed cell differentiation.

### Abnormal Splice Regulation of Genes Important for Glial Cells Maturation is Observed in eIF2B-mutated Brains

In order to further analyse the dysregulation of genes involved in mRNA stabilization and splicing during the CNS development, we focussed our attention on two genes with splice isoforms differently expressed during glial maturation, respectively *PLP1* in oligodendrocytes and *GFAP* in astrocytes.

We found an up-regulation of the ratio *PLP+DM20/PLP* demonstrating an increased expression of the *DM20* isoform specifically in the mutated brains at foetal age (Figure 2). This up-regulation of *DM20* in the eIF2B-mutated foetal brains is concomitantly associated with an increased expression of *HNRNPH1* and *F*. Regulation of *PLP* alternative splicing and maintenance of the *PLP/DM20* ratio are critical for oligodendrocytes differentiation and myelin maintenance. In mature myelinating oligodendrocytes, the *PLP* 5′ splice site is preferentially used, while in oligodendrocytes progenitor cells and in other cell types, *DM20* is the preferred site [23]. Mutations that impair the PLP/DM20 ratio cause a spectrum of dysmyelinating disorders in humans [47], [48]. Therefore, alteration of PLP/DM20 mRNA splicing or isoforms stability related to *HNRNP* dysregulation could account for the myelin paucity with substantial increase in the number of non myelinating oligodendrocytes observed in eIF2B-related disorders [18], [49].

We also found that mutated brains over-express *GFAPdelta* at all the developmental stage but not *pan-GFAP* and *GFAPalpha*. As a consequence, the ratio *pan-GFAP/GFAPdelta* is down-regulated in the children/adult mutated brains, whereas it is not affected in the foetal brains (Figure 2). *GFAPdelta* results from an alternative splicing of *GFAP* gene, which replaces the two final exons of the predominant isoform *GFAPalpha* with an alternative terminal exon [50]. In normal adult grey and WM parenchyma, *GFAPdelta* represents only a small fraction of total *GFAP* [51]. However, *GFAPalpha* and *delta* transcripts are both up-regulated in Alzheimer’s disease [52]. Moreover, the transient over-expression of *GFAPdelt*α in a human astrocytoma cell line, destabilizing the ratio *GFAP alph*α*/delta*, results in the formation of cytoplasmic aggregates that often collapse the endogenous GFAP networks [51]. Similarly to the present study, an over-expression of the mRNA and protein isoform GFAPdelta have been recently reported in the brain WM of an eIF2B-mutated child (4.5 months) and two adult (12 and 29 years) patients compared to control brains without differences in the major GFAPalpha isoform and in the ratio GFAPalpha/pan-GFAP [53]. The abnormal morphology of mutated astrocytes has been invoked to explain the extensive WM cavitation and limited gliosis of affected tissues observed in eIF2B-mutated brains [20]. In the affected WM, astrocytes have coarse blunt processes instead of the fine arborisations observed in controls. In the LD related to *GFAP* mutations (Alexander disease), the inability to form proper astrocytic GFAP networks coexists with incomplete maturation of astrocytes and insufficient myelin formation [54]. Our findings suggest that assembly-compromised GFAPdelta could alter protein interactions of GFAP filaments, thereby contributing to the aberrant morphology of affected astrocytes. This may contribute to the clinical similarity found between eIF2B and GFAP-related disorders, both being a cavitated demyelinating LD with stress-induced acute phases [2].

We described here for the first time genes involved in the mRNA stabilization and splicing machinery as potential actors in the abnormal maturation of glial cells observed in the eIF2B-mutated patient brains. However, if the link between the hnRNP proteins and the abnormal splice of *PLP/DM20* mRNA is clear, the relation between hnRNP and *GFAP* mRNA splicing remain to be elucidated.

EIF2B-mutated fibroblasts lack a specific transcriptomic ER-stress profile but they differentially express genes important for mRNA regulation (HNRNP), mitochondrial metabolism/transport and gonadogenesis when compared to controls. Moreover, the abnormal HNRNP mRNA production observed in eIF2B-mutated brains varies during cerebral developmental and is concomitant with the splice dysregulation of the main genes involved in glial maturation. This suggests that the brain susceptibility of abnormal translation regulation related to eIF2B mutations involves a developmental expression deregulation of proteins implicated in mRNA splicing and stabilization machinery like the hnRNP which play a major role in other neurodegenerative disorders.

## Materials and Methods

### Ethics Statement

Studies have been performed with the ethical agreement of the “Centre de protection des personnes Sud-Est VI”, France and a signed informed consent of the parents. All patients or their legal guardians gave their written informed consents.

### Samples from Fibroblasts of Leukodystrophic Patients

For the differential transcriptomic study, we analysed the leukodystrophic fibroblasts from ten eIF2B-mutated patients and from ten patients affected by OL coupled with 20 sex and age-matched control subjects without neurological signs (Table 1).

In the eIF2B-mutated group, all patients have a severe phenotype characterised by (i) an early infantile age of onset (mean 1.2±0.7 years, range: 5 months to 2.7 years), and (ii) a rapid progression leading to death or absence of motor and cognitive capacities in a mean of 1.7±1.4 years (range: 10 days to 3.5 years) (Table 1). We selected patients with a severe, early infantile form, because the impacts of the eIF2B mutations on the phenotype seem greater than in milder forms in which other environmental factors are also involved [33].

The 10 patients with OL have also an infantile age of onset (mean 0.9±1.3 years, from birth to 3.5 years). They included (i) five patients with other types of vacuolating LD (three patients with Alexander disease relative to *GFAP* mutation and two patients with megalencephalic leukoencephalopathy with subcortical cysts related to *MLC1* mutations), (ii) two patients with a severe congenital form of hypomyelinating LD caused by *PLP1* mutations PMD form 0 without motor acquisition), (iii) three patients with a demyelinating LD related to lipid metabolism enzymatic defect with a rapid progression in one case of Krabbe disease and a slower disease evolution in two cases of SLS (Table 1).

### Samples from Patients’ Brains

Affected brain tissues used for quantitative real-time PCR gene analysis (Tables 4 and 5), provided from the brains samples of two eIF2B-mutated fœtuses (393 and 1767), were obtained after the therapeutic abortions performed at respectively 14 and 16 weeks of gestation due to the affected status of the foetuses found by DNA’CVS analysis and immediately frozen in liquid nitrogen. In addition, brain samples of affected patients were identically frozen immediately after the brain autopsy performed between 13 months and 39 years. Control brain samples (foetus and children/adults) were provided by the Brain and Tissue Bank for Developmental Disorders at the University of Maryland, Baltimore, Maryland (USA) and the fetopathologic unit of the Clermont-Ferrand University Hospital, (Dr AM Beaufrère), Clermont-Ferrand (France).

**Table 5:** List of the genes quantified by QRT-PCR analysis.

qPCR Primers	Sequence (5′-3′)
Total *GFAP*, forward	AGAAGCTCCAGGATGAAACC
Total *GFAP*, reverse	TTCATCTGCTTCCTGTCTATAGG
*GFAPalpha*, forward	AGAGGTCATTAAGGAGTCCA
*GFAPalpha*, reverse	CAACTATCCTGCTTCTGCTC
*GFAPdelta*, forward	CCTACAGGAAGCTGCTAGAG
*GFAPdelta*, reverse	GCGTTCCATTTACAATCTGGT
*GAPDH*, forward	CTCTCTGCTCCTCCTGTTCGAC
*GAPDH*, reverse	TGAGCGATGTGGCTCGGCT
*HPRT*, forward	ATGGGAGGCCATCACATTGT
*HPRT*, reverse	ATGTAATCCAGCAGGTCAGCAA

Transcript-specific primers and their sequences for SYBR Green quantitation are mentioned.

### Cell Culture, Thapsigargin Treatment and RNA Isolation

Primary fibroblasts were obtained from skin biopsy. They were grown on RPMI 1640 L-Glutamine medium (Gibco) supplemented with 1% Penicillin 1000UI/ml, Streptomycin 10mg/ml, 0,001% Amphotericin B 2,5mg/ml and 10% FBS (Foetal Bovine Serum) at 37°C and 5% CO_2_. Twenty-four hours after treatment, fibroblasts at nearly confluence were trypsinized with 0.1% trypsin-EDTA and cells were equally divided. One half of the cells were treated with 1µM thapsigargin and the other half with 0.05% ethanol during 4H at 37°C and were harvested with two PBS washes just after thapsigargin treatment. Thapsigargin raises cytosolic calcium concentration by blocking the ability of the cell to pump calcium into the endoplasmic reticulum which causes these stores to become depleted. Finally, fibroblasts from 6 control subjects used as reference were grown, harvested, lysed and RNA extracted identically to the others. Total RNA from fibroblasts and from all brain samples were extracted using the RNeasy Lipid Tissue Mini Kit (Qiagen), according to the manufacturer’s instructions and stored at −80°C.

### RNA Preparation for Microarray Experiments

For each sample, RNA quality and concentration were assessed using a Bioanalyseur 2100 (Agilent Technologies, Waldbronn, Germany), according to the manufacturer’s instructions. For microarray hybridizations, 500 ng of total RNA were directly labelled by reverse transcription using the Low RNA input linear amplification (Agilent Technologies) and were purified using the RNeasy mini kit (Qiagen) according to the manufacturer’s recommendations. This reaction has been performed for RNA of each fibroblast of eIF2B-mutated patients, OL-patients and age- and sex-matched control subjects with Cy3 (green) incorporation. The reference sample (pool of 6 control subject’s fibroblasts) was labelled with Cy5 (red) incorporation. All the dye incorporation rates were checked by ND-1000 spectrophotometer (Nanodrop Technologies). Fragmentation of cDNA was performed by denaturating the samples at 60°C during 30 min in order to obtain cDNA fragments of 50 to 200 nucleotides.

### cDNA Microarray Hybridizations and Scanning

Two microarray experiments have been performed with and without ER-stress conditions: i) M1: cDNA samples of 10 eIF2B-mutated fibroblasts *versus* matched coupled controls fibroblasts; ii) M2: cDNA samples of 10 OL fibroblasts *versus* matched coupled controls fibroblasts.

750 ng cDNA (M1) or 825 ng cDNA (M2) of mutated and controls cells, treated with thapsigargin or ethanol and labelled respectively with Cy3 and Cy5, were mixed into a single pool with the hybridization buffer. They were cohybridized on the same microarray slide (Human pangenomic 44K from Agilent Technologies) in an Agilent hybridization platform (Imaxio, Diagnogene division, Saint-Beauzire, France) at 65°C during 17h. Microarray slides were then scanned with the following parameters: i) M1 experiment: Cy3 Photo Multiplier Tube (PMT): 100, and Cy5 PMT: 100 with a 10 µM resolution; ii) M2 experiment: parameters for Cy3 and Cy5: Xdr High 100% −Xdr Low 10% with a 5 µM resolution, the Xdr corresponding to two scans (one at PMT 100 and one at PMT 10). These processes were repeated for each of the 40 hybridized slides. The images were analyzed with the Feature Extraction 9.1 software (Agilent Technologies) using the GE2-v4_91 (M1) or GE2-v5_95 (M2) protocols according to the manufacturer’s instructions.

### cDNA Microarray Data Analysis

The statistical analysis was performed for characterized genes. EST and chromosomal location (LOC) have been excluded.

#### Correction and normalization

Transformation and normalization of hybridization data were performed to minimize variations arising from technical differences in RNA quality, probe labelling, and hybridization conditions between experiments. The repartition of the gene expression rates is asymmetric with a small number of high values leading us to perform a logarithmic transformation and standardisation for each signal intensity (giving the “log values”). Correction was next performed for differences in the variability across the range of gene expression levels using the formula : (corrected G log value)i  =  (G log value)i – (R log value)i + (mean R log value)i; where “G log value” represents sample signal intensity, “R log value” represents the reference signal intensity, and “mean R log value” represents the mean of all R values obtained for the gene “i” in reference across the different conditions.

### Identification of Genes Specific of eIF2B-related Disorder

Genes differentially expressed (i) between the eIF2B-mutated patients and the control subjects for the M1 and (ii) between the OLs-patients and the control subjects for the M2, were determined statistically by variance analysis using the GeneANOVA software [32]. For M1 and M2 analyses, we constructed a statistical model including 3 factors: disease status (patients or control subjects), patients’ couple (couple 1 to 10) and treatment (thapsigargin or ethanol). Then we identified genes specific of eIF2B-related disorder by comparing the experiments M1 and M2 and the genes differentially expressed between the eIF2B-mutated patients and the OLs-patients were determined statistically by variance analysis using the GeneANOVA software [32]. We constructed a statistical model including 2 factors: disease status (patients or control subjects) and treatment (thapsigargin or ethanol). The variance across each group of patients was included in residual.

### *In Silico* Analyses of the Biological Processes Involved in eIF2B-related Disorders

Biological processes were analysed using the Genomatix software, available at http://www.genomatix.de. Using the BBSPE (Bibliosphere) function, we analysed and selected biological processes involving a maximum of the 70 selected genes. For each biological process, a z-score (or z-value) is calculated: it expresses the divergence of the experimental result from the most probable result as a number of standard deviations. The larger the value of *z*, the less probable the experimental result is due to chance. When z-score is over 3, one can consider that the result is significantly not due to chance.

### Gene Expression Analysis of Selected Genes by Quantitative Real-time PCR (QRT-PCR)

Total RNA extracted from fibroblasts and from the brain samples were used for gene expression analyses by QRT-PCR of 13 selected genes, as well as *PLP/DM20* and *GFAP* isoforms (Tables 2 and 3). RT for each sample has been performed with 1µg of total RNA using the High Capacity cDNA Reverse Transcription kit (Applied Biosystems) according to the manufacturer’s instructions. Two independent RT were performed for each sample and quantitative PCR was performed on Taqman 7300 using the Taqman Probe technology (Applied Biosystems) with specific probes and primers (Taqman Gene Expression Assay) for each 13 selected genes and *PLP/DM20* isoforms, pre-designed to overlap exon-exon boundaries and then prevent genomic DNA amplification. Quantitation was carried-out regarding the housekeeping *beta2-microglobulin* (*B2M*) mRNA. For each selected gene, 4 µl of 1/10 cDNA dilution were amplified with 1X Taqman Gene Expression Master Mix (Applied Biosystems) and 1X of Taqman Gene Expression Assay (Tables 2 and 3) in a final volume of 20 µl. The program included an initial step of UDG incubation at 50°C for 2 min, a step of enzyme activation at 95°C for 10 min followed by 40 cycles of 95°C for 15 seconds, 60°C for 1 min. The QRT-PCR were carried out in duplicates for each RT sample and each selected gene. The cycle of threshold value (Ct) was used to calculate the relative expression of the gene of interest and normalized to the transcript for the housekeeping gene *B2M*. Expression rate of QRT-PCR were calculated using the formula Mean (2^∧^-(delta(patient)-delta(control))) where delta(patient) correspond to (Ct gene *x* − Ct *B2M*) for one patient and delta (control) to (Ct gene *x* − Ct *B2M*) for the corresponding control.

For the *GFAP* isoforms quantifications, transcript-specific primers were designed to overlap exon-exon boundaries to prevent genomic DNA amplification [53] (Table 5). The PCR was carried out using a sample volume of 20 µl containing Power SYBR Green PCR Master Mix (Applied Biosystems), 10 µM primers and 4 µl of 1/10 cDNA dilution. The relative abundance of transcript expression was calculated using the cycle of threshold value and normalized to the endogenous controls *GAPDH* (*Glyceraldehyde Phosphate Deshydrogenase*) and *HPRT* (*Hypoxanthine-guanine Phosphoribosyl Transferase*). The results were expressed as mean ± S.E.M. and compared using the Mann-Whitney test.

### Protein Extraction and Western Blot Analysis

To assess the expression of hnRNP proteins in the brain autopsy samples, we performed a nuclear and cytoplasmic differential extraction, due to the major nuclear localisation of these proteins, using the NE-PER nuclear and cytoplasmic extraction reagents (Thermo Scientific) according to the manufacturer’s instructions. We validated the fractionation procedure using the anti-trimethyl-histone H3 (Lys27) (H3K27) antibody, that recognizes a histone mark specific to the nucleus. The cytoplasmic fractions were also used for PLP and GFAP detection. Protein concentration was then assessed by the Bradford method. 10 µg of protein lysates were separated on 10% SDS-polyacrylamide gel electrophoresis and transferred to polyvinylidene difluoride membranes (Millipore). Unspecific binding were blocked with 5% bovine serum albumine (BSA) in Tris-buffered saline (TBS) containing 0.1% Tween 20 overnight at 4°C. Incubations with primary antibodies were carried out at room temperature using mouse anti-GAPDH (1∶5000, Abcam, ab8245), mouse anti-hnRNPL (1∶8000, Sigma Aldrich, R4903), mouse anti-hnRNPU (1∶8000 for cytoplasmic fraction and 1∶12000 for nuclear fraction, Sigma Aldrich, R6278), rabbit anti-GFAP (1∶1500, Chemicon, ab5804), rabbit anti-GFAP delta (1∶500, Abcam, ab28926), rat anti-PLP (1∶200, gift from W Macklin) and rabbit anti-H3K27 (1∶200, Millipore, #07-449). After washing, membranes were incubated with an accordingly HRP-conjugated secondary antibody: anti-mouse (1∶5000, Amersham), anti-rabbit (1∶5000, GE Healthcare) or anti-rat (1∶5000, Rockland) at room temperature. Protein expression was detected by ECL reagent (GE Healthcare).

Bands on the films were quantified with ImageQuant TL software and compared using the Mann-Whitney test.

## Supporting Information

Table S1 **List of the 70 genes specifically dysregulated in eIF2B-mutated Fb obtained by microarray analysis.**
(DOC)
