# Supplementary material for: Developmental Splicing Deregulation in Leukodystrophies Related to EIF2B Mutations
Source: PLoS One. 2012 Jun 21;7(6):e38264. doi: 10.1371/journal.pone.0038264 (PMC3380860; doi:10.1371/journal.pone.0038264)
Supplement: Table S1 — List of the 70 genes specifically dysregulated in eIF2B-mutated Fb obtained by microarray analysis. (DOC) [file pone.0038264.s001.doc]

# TABLE S1

**List of the 70 genes specifically dysregulated in eIF2B-mutated Fb obtained by microarray analysis.**

| **Refseq mRNA** | **Gene Name** | **Description** | **FDR (%)** | **Ratio CACH/VWM / Healthy** |
| --- | --- | --- | --- | --- |
| NM_005520 | *HNRNPH1* | Heterogenous nuclear ribonucleoprotein H1 | 0 | 0,66 |
| NM_001533 | *HNRNPL* | Heterogenous nuclear ribonucleoprotein L | 0 | 0,77 |
| XR_017206 | *LOC643744* | Similar to D-PCa-2 protein isoform c | 0 | 0,73 |
| NM_005517 | *HMGN2* | High-mobility group nucleosome-binding domain 2 | 0 | 0,67 |
| NM_002710 | *PPP1CC* | Protein phosphatase 1C catalytic subunit | 0 | 0,72 |
| NM_031844 | *HNRNPU* | Heterogenous nuclear ribonucleoprotein U | 0 | 0,75 |
| NM_004965 | *HMGN1* | High-mobility group nucleosome binding domain 1 | 0 | 0,79 |
| NM_006265 | *RAD21* | Protein involved in DNA double-strand break repair | 0 | 0,64 |
| NM_182800 | *ARS2* | Arsenite resistance protein 2 | 0 | 0,78 |
| NM_007375 | *TARDBP* | TAR DNA binding protein | 0,01 | 0,80 |
| NM_004521 | *KIF5B* | Kinesin family member 5B | 0,01 | 0,86 |
| NM_020810 | *TRMT5* | tRNA methyltransferase 5 homolog (S. cerevisiae) | 0,01 | 0,73 |
| NM_017824 | *MARCH5* | Membrane associated ring finger (C3HC4) 5 | 0,01 | 0,86 |
| AY374131 | *MTHFD1L* | Methylenetetrahydrofolate dehydrogenase (NADP+ dependent) 1-like | 0,01 | 0,80 |
| NM_005333 | *HCCS* | Holocytochrome c synthase | 0,01 | 0,84 |
| NM_022051.1 | *EGLN1* | egl nine homolog 1 (C.elegans) | 0,01 | 0,84 |
| NM_014623 | *MEA1* | Male-enhanced antigen 1 | 0,01 | 0,80 |
| NM_025165 | *ELL3* | Elongation factor RNA polymerase II-like 3 | 0,01 | 1,07 |
| NM_006072 | *CCL26* | Chemokine (C-C motif) ligand 26 | 0,01 | 1,06 |
| NM_022913 | *GPBP1* | GC-rich promoter binding protein 1 | 0,02 | 0,84 |
| NM_003677 | *DENR* | Density-regulated protein | 0,02 | 0,80 |
| NM_006559 | *KHDRBS1* | KH domain containing, RNA binding, signal transduction associated 1 | 0,02 | 0,70 |
| NM_005826 | *HNRNPR* | Heterogenous nuclear ribonucleoprotein R | 0,02 | 0,77 |
| NM_002539 | *ODC1* | Ornithine decarboxylase 1 | 0,02 | 0,68 |
| NM_000709 | *BCKDHA* | Branched chain keto acid dehydrogenase E1, alpha polypeptide | 0,02 | 0,85 |
| NM_005614 | *RHEB* | Ras homolog enriched in brain | 0,02 | 0,86 |
| NM_021038 | *MBNL1* | Muscleblind-like 1 (Drosophila) | 0,02 | 0,74 |
| NM_022662 | *ANAPC1* | Anaphase promoting complex subunit 1 | 0,03 | 0,77 |
| AK023110 | *SSU72* | SSU72 RNA polymerase II CTD phosphatase homolog (S. cerevisiae) | 0,04 | 0,83 |
| NM_014426 | *SNX5* | Sorting nexin 5 | 0,04 | 0,75 |
| NM_003200 | *TCF3* | Transcription factor 3 (E2A immunoglobulin enhancer binding factors E12/E47) | 0,04 | 0,79 |
| NM_031314 | *HNRPC* | Heterogenous nuclear ribonucleoprotein C | 0,04 | 0,78 |
| NM_001007553 | *CSDE1* | Cold shock domain containing E1, RNA-binding | 0,04 | 0,87 |
| NM_003348 | *UBE2N* | Ubiquitin-conjugated enzyme 2N | 0,04 | 0,83 |
| NM_014984 | *AZI1* | 5-azacytidine induced 1 | 0,04 | 0,77 |
| NM_031370 | *HNRNPD* | Heterogenous nuclear ribonucleoprotein D | 0,04 | 0,75 |
| NM_006601 | *PTGES3* | Prostaglandin E synthase 3 (cytosolic) | 0,04 | 0,85 |
| NM_032478 | *MRPL38* | Mitochondrial ribosomal protein L38 | 0,05 | 0,82 |
| NM_025128 | *MUS81* | MUS81 endonuclease homolog (S.cerevisiae) | 0,05 | 0,83 |
| NM_012325 | *MAPRE1* | Microtubule-associated protein, RP/EB family, member 1 | 0,05 | 0,85 |
| NM_014184 | *CNIH4* | Cornichon homolog 4 (Drosophila) | 0,05 | 0,87 |
| NM_005662 | *VDAC3* | Voltage-dependent anion channel 3 | 0,05 | 0,82 |
| NM_001681 | *ATP2A2* | ATPase, Ca++ transporting, cardiac muscle, slow twitch 2 | 0,05 | 0,83 |
| NM_003146 | *SSRP1* | Structure specific recognition protein 1 | 0,05 | 0,81 |
| NM_139313 | *YME1L1* | YME1L-like 1 (S.cerevisiae) | 0,05 | 0,84 |
| AK125922 | *CSNK2A2* | Casein kinase 2, alpha prime polypeptide | 0,06 | 0,86 |
| NM_003002 | *SDHD* | Succinate dehydrogenase complex, subunit D, integral to membrane protein | 0,06 | 0,85 |
| NM_014670 | *BZW1* | Basic leucine zipper and W2 domains 1 | 0,06 | 0,73 |
| NM_144778 | *MBNL2* | Muscleblind-like 2 (Drosophila) | 0,06 | 0,67 |
| NM_030811 | *MRPS26* | Mitochondrial ribosomal protein S26 | 0,07 | 0,86 |
| NM_006428 | *MRPL28* | Mitochondrial ribosomal protein L28 | 0,07 | 0,86 |
| NM_003798 | *CTNNAL1* | Catenin (cadherin-associated protein), alpha-like 1 | 0,07 | 0,61 |
| NM_004279 | *PMPCB* | Peptidase (mitochondrial processing) beta | 0,07 | 0,83 |
| NM_013438 | *UBQLN1* | Ubiquilin 1 | 0,07 | 0,89 |
| NM_006331 | *EMG1* | EMG1 nucleolar protein homolog (S.cerevisiae) | 0,07 | 0,79 |
| NM_017840 | *MRPL16* | Mitochondrial ribosomal protein L16 | 0,08 | 0,85 |
| NM_017755 | *NSUN2* | NOL1/NOP2/sun domain family, member 2 | 0,08 | 0,83 |
| NM_004523 | *KIF11* | Kinesin family member 11 | 0,08 | 0,77 |
| NM_182640 | *MRPS9* | Mitochondrial ribosomal protein S9 | 0,08 | 0,83 |
| NM_001031740 | *MANEAL* | Mannosidase, endo-alpha-like | 0,09 | 1,07 |
| NM_001008735 | *HMG1L1* | High-mobility group (nonhistone chromosomal) protein 1-like 1 | 0,09 | 0,71 |
| NM_018225 | *SMU1* | Smu-1 suppressor of mec-8 and unc-52 homolog (C.elegans) | 0,09 | 0,84 |
| NM_003286 | *TOP1* | Topoisomerase (DNA) 1 | 0,09 | 0,76 |
| NM_001419 | *ELAVL1* | ELAV (embryonic lethal, abnormal vision, Drosophila)-like 1 (Hu antigen R) | 0,1 | 0,86 |
| NM_000175 | *GPI* | Glucose phosphate isomerase | 0,1 | 0,75 |
| NM_198329 | *UBE1DC1* | Ubiquitin-activating enzyme E1-domain containing 1 | 0,1 | 0,79 |
| NM_002823 | *PTMA* | Prothymosin, alpha | 0,1 | 0,70 |
| NM_005782 | *THOC4* | THO complex 4 | 0,1 | 0,80 |
| NM_001790 | *CDC25C* | Cell division cycle 25 homolog (S.pombe) | 0,11 | 0,87 |
| NM_030932 | *DIAPH3* | Diaphanous homolog 3 (Drosophila) | 0,12 | 0,84 |

FDR : False Discovery Rate

Expression rate was calculated using the formula Mean ((Corrected G log value)i/(Corrected G log value)j) were (Corrected G log value) are calculated as described in Material and Methods. i correspond to one patient’s fibroblast sample and j to one control’s fibroblast sample.
